# Role of Personality in Behavioral Responses to New Environments in Captive Asiatic Lions* (Panthera leo persica)*

**DOI:** 10.1155/2017/6585380

**Published:** 2017-05-30

**Authors:** Giovanni Quintavalle Pastorino, Anna Viau, Giulio Curone, Paul Pearce-Kelly, Massimo Faustini, Daniele Vigo, Silvia Michela Mazzola, Richard Preziosi

**Affiliations:** ^1^Institute of Zoology, Zoological Society of London, Regents Park, London NW1 4RY, UK; ^2^Department of Veterinary Medicine, Università degli Studi di Milano, Via Celoria 10, 20133 Milan, Italy; ^3^The Royal Veterinary College, Royal College Street, London NW1 0TU, UK; ^4^Division of Biology and Conservation Ecology, School of Science and the Environment, Faculty of Science and Engineering, Manchester Metropolitan University, Manchester M1 5GD, UK

## Abstract

Studying personality in captive animals may enable the development of individual-based management decisions, which may improve animal welfare. Asiatic lions at London Zoo represent an opportunity to research an understudied species' response to new environments since they have experienced social and physical changes, such as new enclosures and increased social interaction with humans. This project aimed to investigate the role of personality in behavioral responses to these changes. Lion personality questionnaires completed by keepers and direct focal animal observations were used to create personality profiles. Time budgets and enclosure use were determined and compared between control nights and event nights and between the lions' previous enclosure and their new one. The results showed a lack of difference in time budget and enclosure use between control and social event nights, and the spread of participation index values revealed that the lions use their enclosures unevenly. Personality profiles identified various traits that could assist with individual-based management decisions. As the first study to assess Asiatic lions personality, this research contributes to the creation of consistent and valid methodology for evaluating captive animal personality that may improve husbandry and welfare protocols for individual lions, leading to the improved health and success of the species.

## 1. Introduction 

Animal personality research has continually developed since Pavlov's studies on dogs [1]. Despite this, an array of terminology and methodology exists. Several alternative words to personality have been used, including “individual behavioral variation” [2] and “temperament” [3]. Recently, there has been a push to generate common methodologies such that research would be comparable across species and locations. To help attain this goal, personality has been defined as “behavioral differences across individuals that are consistent over time and across contexts or situations” [4].

Previous studies have demonstrated that animal personality is measureable, has a degree of cross-species comparability, and can be assessed in various species, including mammals, fish, and insects [5]. Originally, research focused on using animals as models for human personality [6]. However, animal personality research has progressed, allowing for a unique approach to welfare concerns, conservation issues, and reintroduction success [7–9].

Practical applications of personality research in captive animals include husbandry, training, and breeding programs. Personality assessment facilitates more individual-based management, which may help to maximize the welfare and overall success of a captive collection [10, 11]. For example, Chadwick [7] found that personality assessments of cheetahs* (Acinonyx jubatus)* helped zoos organize successful breeding groups and that pairs with more divergent personalities had greater breeding success. Carlstead et al. [12] found similar results in their study on black rhinos* (Diceros bicornis)*. Improved breeding success enhances welfare by decreasing the need for relocating animals because a mismatched pair had low reproductive success.

Prior to introducing an animal to a captive collection, personality assessment can help to determine how the new individual will affect group dynamics [10]. This method can also be applied in reintroduction programs to predict an animal's response to release into a new location [13]. For instance, some individuals have personality traits (e.g., boldness) that may cause them to respond inappropriately in stressful or dangerous circumstances [14].

Animal personality is evaluated using various methods, including coding personality traits based on observations of natural behavior or a specific test (e.g., novel object test), and trait rating completed by keepers [8, 11, 15]. Personality questionnaires completed by keepers may give the impression that they are subjective, but previous research has demonstrated that this method is reliable and that results relate to the animal's behavior. Although behavioral observations take considerable time and effort, they are frequently used to create personality profiles as they provide a large amount of reliable data [11, 16]. Multiple methods are often used to validate the profiles, which is essential because the results of the different methods do not always align [8, 12, 16]. For instance, Carlstead et al. [12] found that personality profiles created from keeper-completed questionnaires corresponded to personality assessments created from behavioral observations in black rhinos, but Marieke Cassia and David [17] found that the results of these assessments did not agree in their research on snow leopards* (Uncia uncia)*.

Because of possible discordance, it is important to test additional tools to validate personality profiles. The novel object test, which evaluates an animal's response to new objects (e.g., traffic cone), is commonly used for this purpose [12, 15, 17]. Other tools that could be useful for trait validation include sociograms [16] and the spread of participation index (SPI) [18, 19]. Sociograms are social network diagrams that demonstrate the strength of the relationship between two individuals. SPI produces a value which indicates evenness of enclosure use. By using various methods, appropriate methodology can be created, tested, and shared, enabling the comparison of personality profiles and behavioral responses to new environments across captive collections.

The Asiatic lions* (Panthera leo persica)* at London Zoo have experienced new social and physical environments throughout the last few years. The three females (Rubi, Heidi, and Indi) have moved twice in two years during the construction of their new enclosure at London Zoo, Land of the Lions. A male lion, Bhanu, moved to London Zoo in March 2016. The lions experienced increased human interaction with the opening of the Gir Lion Lodges next to their enclosure and the onset of Sunset Safari evening social events at the zoo during June and July. Other research has documented captive animals' individual responses to new environments [12, 20]. The results of a literature search indicate this is the first study to evaluate Asiatic lion personality. Few studies have been published on felid personality, and most focus on domestic cats [7, 10, 15].

The Asiatic lion is a lion subspecies that resides in Gujarat, India, and is listed as Endangered by IUCN [21]. Although once near extinction, the wild population has been growing steadily due to increased conservation efforts. As of 2015, the Asiatic lion Census estimated the wild population to be approximately 523 individuals [22]. Considering the small wild population, captive Asiatic lion research provides valuable insight into the species' biology and behavior. Captive breeding programs, such as at London Zoo, allow for maximization of the species' genetic diversity and, should the need arise, provide individuals for supplementation of wild populations [23].

The social and physical changes experienced by the lions guided the development of this study, which aims to evaluate the role of lion personality in their behavioral responses to new environments. This study hypothesizes that personality traits identified from keeper questionnaires and observation data create reliable profiles that associate with individual lion behavioral responses to new physical and social environments. Therefore, because of individual personality variation, this study also hypothesizes that these new environments will alter individual time budgets and enclosure use. To test these hypotheses, previously collected behavioral data (i.e., time budget and enclosure use) from Whipsnade Zoo were compared with data from their new enclosure at London Zoo. These data were also compared between control nights and Sunset Safaris. Considering this behavioral data, personality profiles were constructed to determine if certain traits are associated with individual lion responses to new environments. A sociogram was constructed to determine if the relationships between the lions are impacted by their individual personalities.

This study can be considered a case study that may be used to improve the management of these four individuals. Furthermore, this research has wider implications for management of the species, in terms of husbandry, enclosure design, health, welfare, and breeding program success. As of December 2015, there were approximately 359 Asiatic lions in captivity (Srivastav, 2016, pers. comm.). Therefore, a study on four animals can provide essential captive lion behavior and personality data, which can be applied in other collections around the world.

## 2. Methods

### 2.1. Study Area and Subjects

The Asiatic lion pride at London Zoo consists of three females and one male (Table 1). The study took place at Land of the Lions, the recently expanded lion enclosure at London Zoo. The females moved into Land of the Lions in February, 2016, from their temporary enclosure at Whipsnade Zoo, and Bhanu arrived in March, 2016, from Winnipeg, Canada. Except for a few brief introductions, the females and Bhanu were kept in separate areas of the enclosure.

### 2.2. Observation Data

Data collection took place from May 31 to July 19, 2016. Focal animal behavioral observations using continuous sampling were completed to record the state and event behaviors at one minute intervals for each animal [24]. Observations were separated into three categories: daytime, control night, and Sunset Safari. Sunset Safaris occurred on Friday evenings from 6 to 10 pm, during which visitors could enjoy food, drink, and performances while exploring the zoo. Daytime observations took place between 8 am and 5 pm on Tuesdays and Fridays, followed by the respective control night and Sunset Safari observations from 6 to 9 pm.

Each 60-minute observation period was divided such that 15 minutes were spent observing each animal. An observation session ended if the focal animal spent five consecutive minutes out of the observer's sight (e.g., indoor). Total observation time summed between observation periods was approximately 87 hours. Included in each observation period were recordings of weather (i.e., sunny, cloudy, or rainy), temperature (https://weather.com, 2016), approximate crowd size, and decibel readings at five minute intervals. Individual lion identification was facilitated by assistance from keepers during the pilot study and by use of binoculars to note specific markings on each individual.

The behaviors recorded followed a standardized felid ethogram compiled by Stanton et al. [25], which was adapted for this project based on behaviors observed during a pilot study and on an ethogram constructed by Joslin [26]. To create time budgets, similar behaviors were put into classes (Table 2), based on groups in similar research [27, 28]. Times when the lions were out of the observer's sight were not included in the time budgets because they did not have access to their indoor area during most observation sessions, so being out of sight was not a possibility. A full ethogram is provided in Table 10.

The London Zoo enclosure was divided into 27 zones to distinguish areas that may be used for different purposes. Twenty-one zones were located in the females' section of the enclosure and six in the male's section. The Whipsnade Zoo enclosure consisted of eight zones. These zones were assigned so that an animal's specific location could be recorded during each observation, which was used to determine each lion's enclosure use for each observation period. Maps of the London and Whipsnade Zoo enclosures and zone descriptions are available in Figures 12 and 13 and Tables 11 and 12.

The spread of participation index (SPI) was calculated to determine evenness of enclosure use. SPI was developed as described by Plowman [18] to allow for zones of unequal areas. Enclosure blueprints provided the areas of Land of the Lions (2195 m^2^) and the enclosure at Whipsnade Zoo (230 m^2^). Possible SPI values range from 0 (even use of the enclosure) to 1 (uneven use of the enclosure). The calculation for SPI is

(1) $$\begin{array}{c} SPI=\frac{\sum \left|f_{o}-f_{e}\right|}{2\left(N-f_{e\;min}\right)}, \end{array}$$

where

 
*f*_*o*_ is the observed frequency of an animal in a zone;
 
*f*_*e*_ is the expected frequency of an animal in a zone;
 
∑|*f*_*o*_ − *f*_*e*_| is the sum of the absolute value of the difference between *f*_*o*_ and *f*_*e*_ for all zones;
 
*f*_*e* min_ is the expected frequency of an animal in the smallest zone;
 
*N* is the total number of observations of an animal in all zones.

London Zoo time budgets and enclosure use were compared to Whipsnade Zoo data, which was collected using the same methodologies in 2015. The data were also compared between Sunset Safaris and control nights. A sociogram was constructed showing the strength of relationships between individuals using time spent in proximity of another lion (i.e., at body-length or nearer). This was completed by calculating Association Index (AI) values for each relationship, as used by Schaller [27] and described by Rees [19]. Possible AI values range from 0 (never seen in proximity) to 1 (always seen in proximity).

(2) $$\begin{array}{c} Association\;\;index=\frac{2N}{n_{1}+n_{2}}, \end{array}$$

where

 
*N* is the number of times lions 1 and 2 were seen together (including when around the third lion);
 
*n*_1_ is the total number of times lion 1 was seen (whether alone or with other lions);
 
*n*_2_ is the total number of times lion 2 was seen (whether alone or with other lions).

### 2.3. Personality

Personality profiles were compiled using questionnaires completed by seven London and Whipsnade Zoo keepers in 2015. The methodology for these questionnaires was adapted from Chadwick's research on cheetah personality [7]. Questionnaires listed 22 traits, which were rated on a scale of 1 (trait was never exhibited) to 12 (trait was always exhibited) by the keepers for each lion. Recent research using these questionnaires led to more traits being added to Chadwick's questionnaire, such as “Friendly to unfamiliar people” [28].

Behaviors recorded during observations were coded similar to time budgets such that classes could be compared to some of the traits on the personality questionnaire (Table 3). Behavioral classes follow those used in similar studies [2, 7, 15]. Profiles created from questionnaires were compared with profiles compiled from observation data. Not all traits were comparable between profiles because only behaviors representing some traits were observed during this study.

### 2.4. Statistical Analysis and Data Presentation

Data analysis was completed using Microsoft Excel 2013 and IBM Statistical Package for the Social Sciences. Due to a small sample size, most tests for statistical significance were deemed inappropriate and therefore analysis focuses on descriptive statistics. Interrater reliability was calculated for the personality questionnaires using intraclass correlation ICC (3, *k*) for the reliability of the mean ratings of the raters [29].

## 3. Results

Bhanu spent little time in his outdoor enclosure during the study because he was still adapting to the enclosure, which totaled to only a few minutes of observation data. Therefore, he was not included in data analysis.

### 3.1. Time Budgets

**Figure 1:**
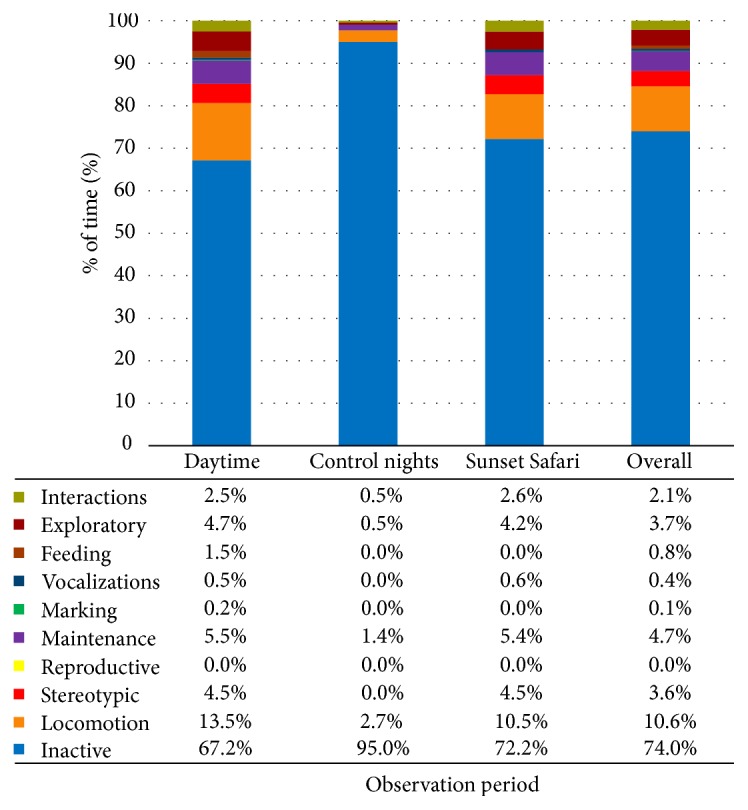
Rubi's time budget for each observation period and overall at London Zoo. Data values are included to show exact percentages of time for each behavior class.

**Figure 2:**
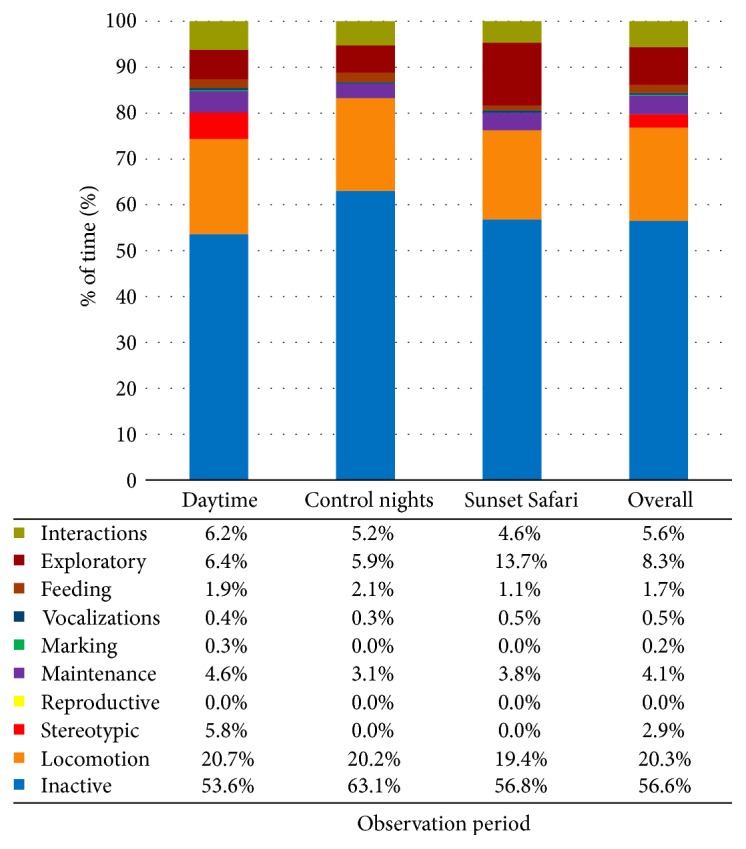
Heidi's time budget for each observation period and overall at London Zoo. Data values are included to show exact percentages of time for each behavior class.

**Figure 3:**
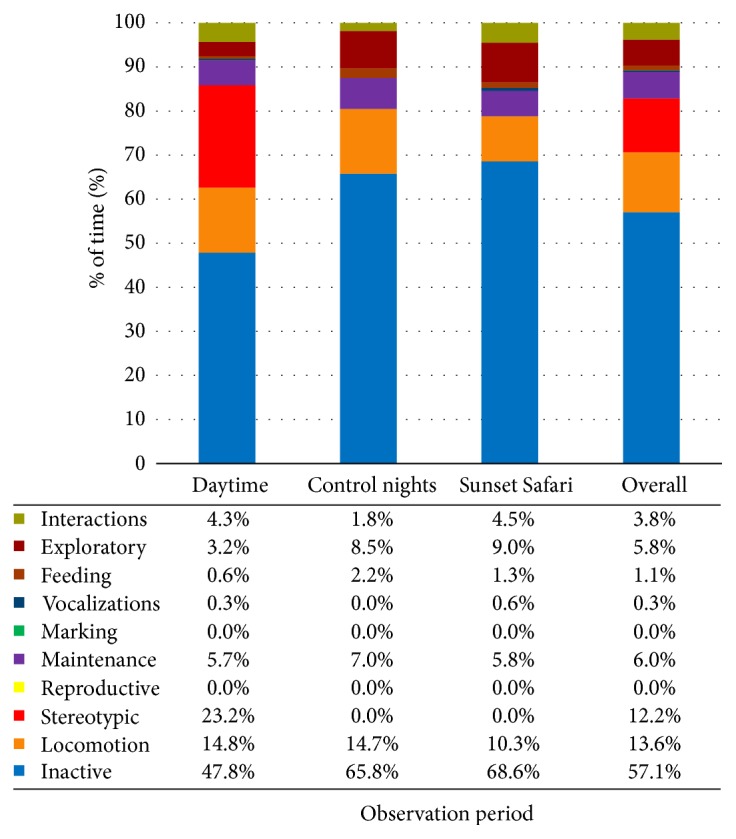
Indi's time budget for each observation period and overall at London Zoo. Data values are included to show exact percentages of time for each behavior class.

The females' time budgets were calculated for each observation period. These were also combined to create overall time budgets for each observation period and in total for all observations. The charts, including data values, are displayed in Figures 1–3.

*Rubi*. See Figure 1.

*Heidi*. See Figure 2.

*Indi*. See Figure 3.

**Figure 4:**
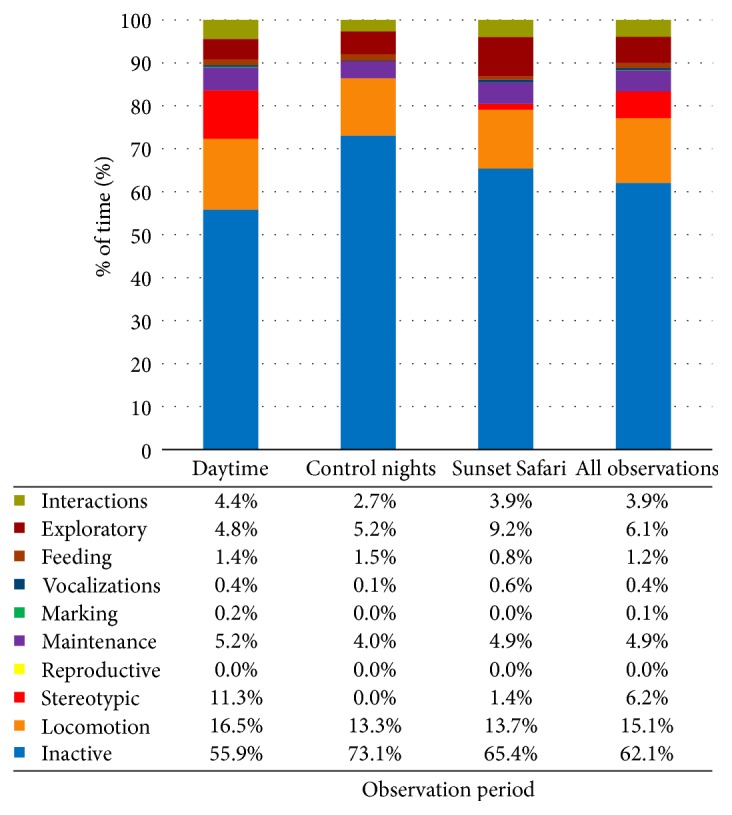
Overall time budgets for each observation period and a complete time budget for all observations at London Zoo. Data values are included to show exact percentages of time for each behavior class.

*Overall*. The chart in Figure 4 shows the overall time budget for each observation period (all females combined) and the time budget for all observations.

**Figure 5:**
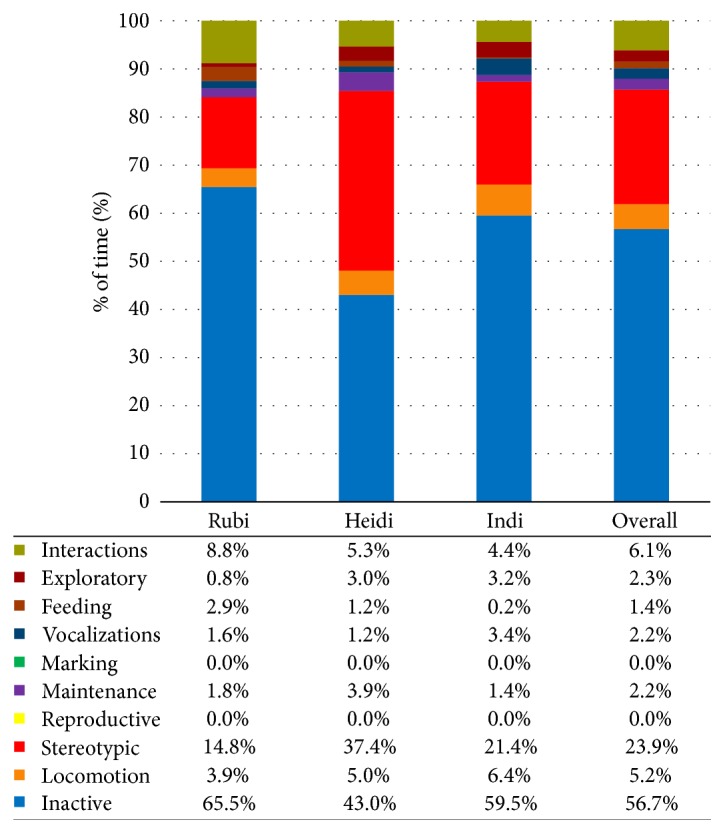
Time budgets for each female for Whipsnade Zoo in 2015. Data values are included to show exact percentages of time for each behavior class.

*Whipsnade Zoo*. Displayed in Figure 5 are time budget data for each female while they were at Whipsnade Zoo.

### 3.2. Enclosure Use

Similar to time budgets, the females' enclosure use was calculated for each observation period and for all observations (Table 4). The 21 zones in the females' section of the enclosure are included; Zones 7–10 are located in the indoor dens and were not included in this study.

**Figure 6:**
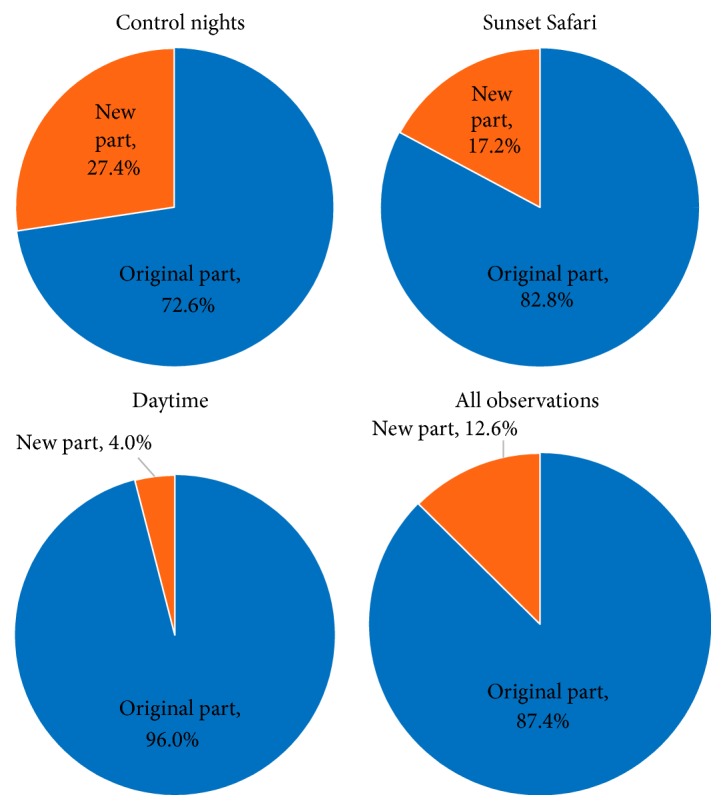
General enclosure use for each observation period and for all observations.

*Overall by Observation Period*. Due to the number of zones, simplified charts are also shown for each observation period's overall enclosure use (Figure 6), which combine zones into two categories: the original part of the enclosure (Zones 1–18) and the new part (Zones 19–25).

**Figure 7:**
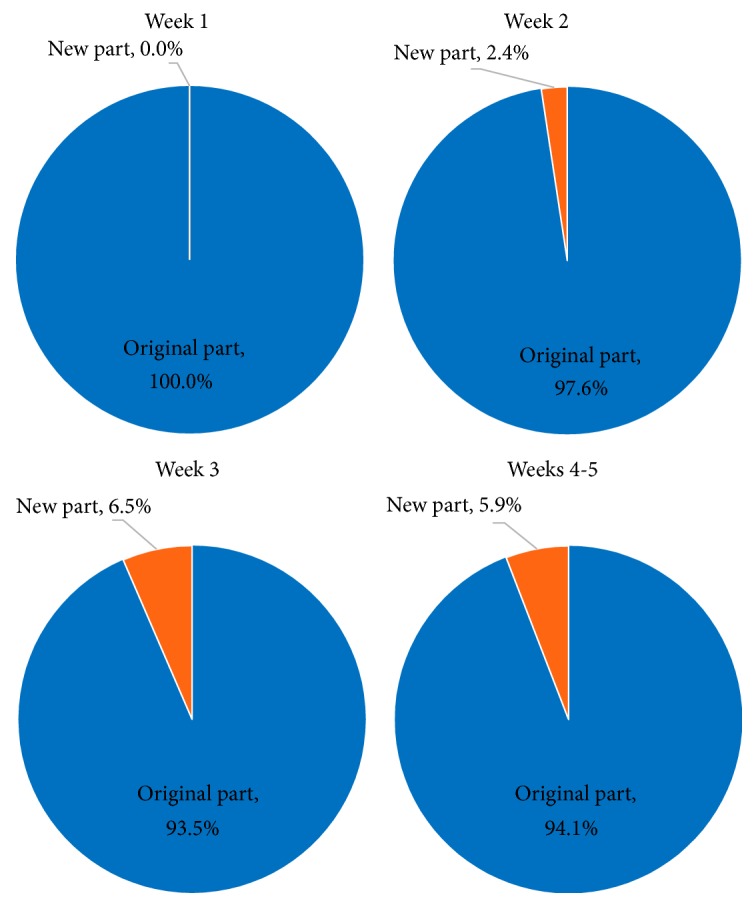
General weekly enclosure use (daytime observations) to compare how the females' enclosure use changed throughout the study.

*Weekly Comparison*. Shown in Table 5 are the enclosure use values combined for the three females categorized by week of daytime observations to show the change in enclosure use over time. To make this easier to visualize, also shown for each week's enclosure use are simplified charts that combine zones into the original part of the enclosure and the new part (Figure 7).

*Whipsnade Zoo*. Whipsnade Zoo enclosure use, for each female and overall, is shown in Table 6.

*SPI*. SPI values for Whipsnade Zoo and London Zoo are displayed in Table 7.

### 3.3. Decibel Levels

Decibel levels were averaged for each observation period and are displayed in Table 8.

### 3.4. Sociality

**Figure 8:**
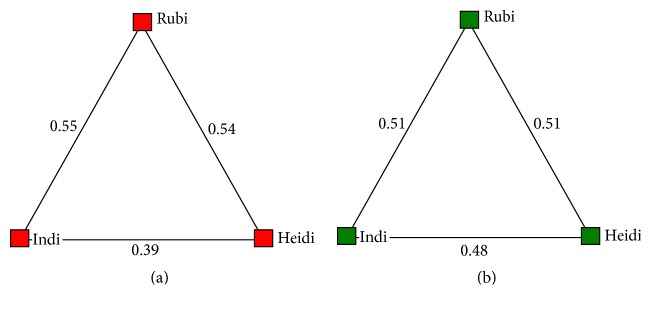
Sociograms displaying the Association Index values for the relationship between the lions at Whipsnade Zoo (a) and London Zoo (b).

Although sociograms are generally used for larger groups of animals, one is provided here for both Whipsnade Zoo and London Zoo to allow for visualization of the AI values and the strength of the relationships between the lions (Figure 8).

### 3.5. Personality

Personality questionnaires were completed in 2015 by seven keepers who worked with the lions at Whipsnade Zoo or London Zoo (Table 9).

Personality questionnaires for each female were highly reliable. For Rubi, the average measure intraclass correlation (ICC) was .761 with a 95% confidence interval from .577 to .886. For Heidi, the average measure ICC was .805 with a 95% confidence interval from .652 to .907. For Indi, the average measure ICC was .857 with a 95% confidence interval from .744 to .932. According to a published interpretation scale [29], these are excellent ICC values.

**Figure 9:**
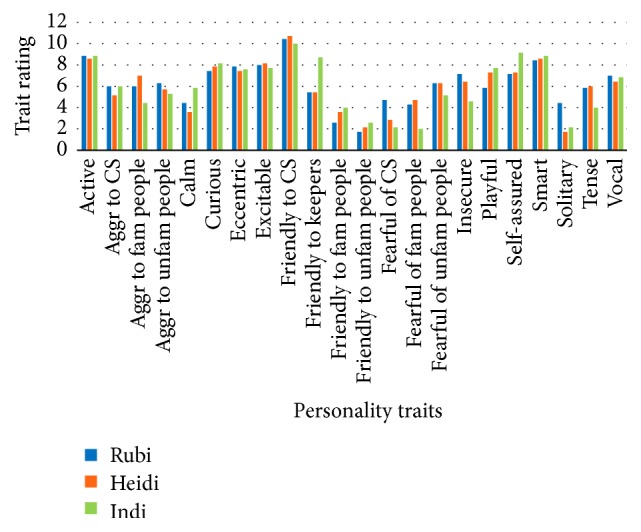
Personality profiles for each female compiled from questionnaires completed by keepers. Aggr = Aggressive, Fam = Familiar, Unfam = Unfamiliar, and CS = Conspecific.

**Figure 10:**
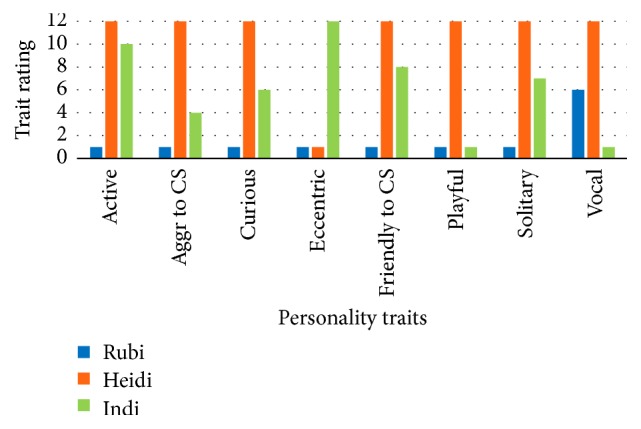
Personality profiles for each female compiled from observation data. Aggr = Aggressive, CS = Conspecific.

**Figure 11:**
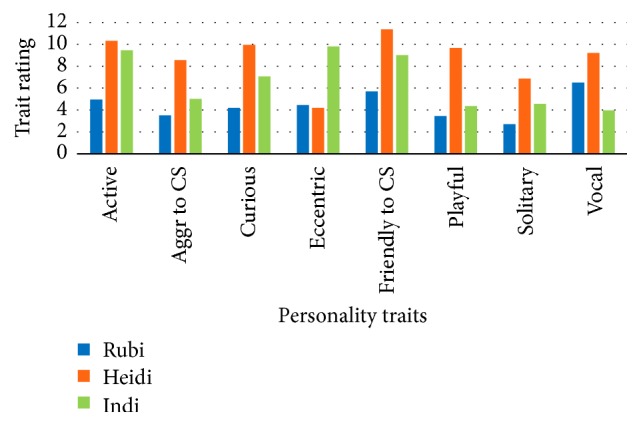
Personality profiles compiled from both keeper questionnaires and observation data for each female. Aggr = Aggressive, CS = Conspecific.

**Figure 12:**
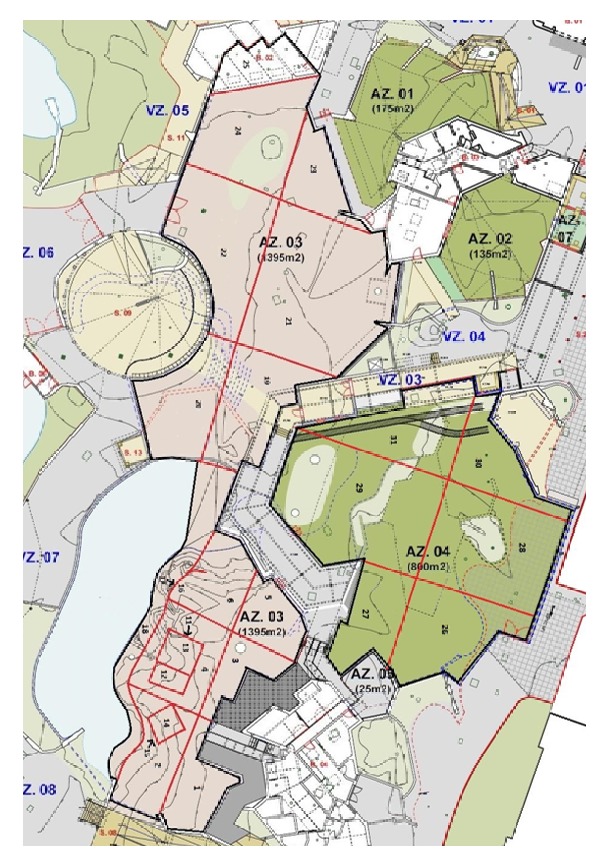
Enclosure map of London Zoo.

**Figure 13:**
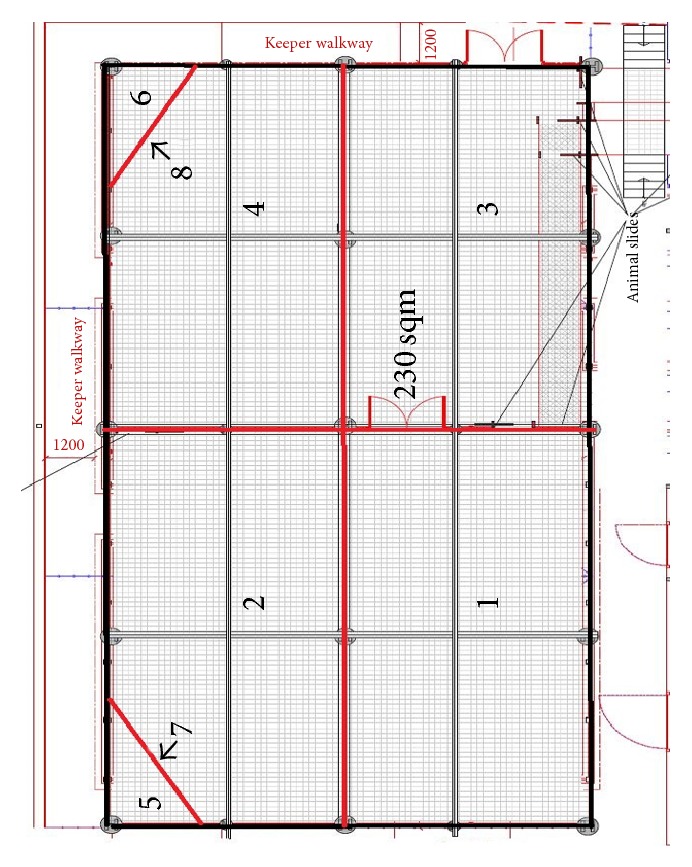
Enclosure map of Whipsnade Zoo.

Personality profiles for each female as determined by the questionnaires and behavioral observations are portrayed separately and combined in Figures 9–11. The charts in Figures 10 and 11 are limited to eight traits as only behaviors fitting into those eight categories were observed. When considering Figure 10, the ratings for each female for each trait are relative to each other.

## 4. Discussion

### 4.1. Time Budget

Overall, the lionesses were inactive for the majority of the time (56.7%), with Rubi displaying the most inactivity (74.0%). The high percentage of inactivity is appropriate as wild lions can sleep up to 21 hours per day [27]. Indi displayed the most stereotypic behavior, which all occurred during daytime observations. However, the frequency of pacing decreased throughout the study. This could be due to a number of factors, such as gradual adjustment to the new enclosure or changes in enrichment or training practices. Previous research found that provision of new objects may lead to a reduction of stereotypic behavior [30, 31].

Observed stereotypic behavior decreased from Whipsnade Zoo to London Zoo. Combining the females' daytime data, stereotypies comprised 24% of the Whipsnade time budget, while only 11% of the London time budget. In part, this may be related to the difference in enclosure size. Lyons et al. [32] suggest that cats kept in smaller enclosures paced more often than cats in larger enclosures. The females' London Zoo enclosure, at 1395 m^2^, is notably larger than their Whipsnade Zoo enclosure (230 m^2^), which may related to the decrease in stereotypic behavior.

Most of the lions' pacing at London Zoo occurred along a chain link fence on the edge of Zone 2. Lyons et al. [32] found that pacing occurred significantly more often along enclosure edges than in other areas. Other studies discussed that areas with fences, through which felids can see conspecifics, other animals, or humans, were associated with increased stereotypic behavior [33, 34]. When the lions moved into the new enclosure, they could see the public on the walkway above this pacing location. The zoo eventually covered this area in an attempt to lessen the occurrence of stereotypies, but the lions may have already established a pacing routine. The lions also paced in front of a metal gate that divided enclosure sections, through which they could sometimes see Bhanu, but observations of this behavior were rare.

Much of the observed pacing behavior occurred before or after their morning feed. The postfeed stereotypic behavior could be due to a short feeding period, which implies that appetite behaviors were not fully expressed [35]. Consequently, the pacing may have been a method to release frustration. Conversely, the stereotypies may originate from a previous stimulus and therefore be independent of any of the lions' experiences at this enclosure [36]. Before the renovation of their London Zoo enclosure, the lions paced in a different area of Zone 1. According to Mason [36], once stereotypies become a part of an animal's behavioral repertoire, their correlation with poor welfare may decrease over time. Therefore, the current pacing may be a “scar” from previous traumatic experiences [36]. Because of this, the stereotypies observed during this study should not be exclusively considered a sign of current welfare concerns without further investigation into the possible causes of this behavior. Frequent use of varied enrichment may decrease pacing behavior in the lions, especially if the methods used are tailored to the needs or preferences of the individual lion [35].

Although lions are typically most active during morning and evening hours [25], the females were more inactive during both evening observation periods than during daytime observations. This could be due to daytime observations starting at 8 am, when the lions tended to be active. In the middle of the day, the lions spent the majority of their time resting. Additionally, some of their daytime activity took place during training or scatter feeds, which would affect the amount of inactivity exhibited when left to behave naturally.

Time budgets varied little between control nights and Sunset Safaris. The lions were inactive for the majority of their time during Sunset Safaris, even with higher overall decibel levels during these events. Rubi and Heidi displayed more activity during Sunset Safari than control nights, but that may have been affected by multiple scatter feeds during one Sunset Safari and the addition of multiple new forms of enrichment to the enclosure before a separate Sunset Safari. This small difference in behavior displayed between observation periods is likely a positive indicator of adjustment to the evening social events. One instance of aggression toward the public occurred during the first Sunset Safari, in which Heidi banged on the glass in front of large group of visitors, at least one of whom was a young child. However, this was the only occurrence of aggression toward the public observed during Sunset Safaris.

### 4.2. Enclosure Use

During daytime observations, the lions spent most of their time in Zones 1-2, where they were mainly inactive. They likely designated these zones as the core area of their territory, in which they felt most secure, with the rest of the enclosure being used for other purposes (e.g., playing, exploration, and occasionally resting) [36]. The lions spent a lot of time in this area before the enclosure was renovated, so their memory of this location may have influenced its current use.

The lions also spent a large portion of their time in Zones 12-13, which make up a raised platform in the original part of the enclosure. These zones gave them a higher viewpoint of the visitor areas and surrounding animal enclosures. Lyons et al. [32] found that big cats often used areas with higher viewpoints for resting and observing. Because the females spent a much of their time on this platform, the addition of a similar structure in the new part of the enclosure might increase the amount of time they spend in that area.

When considering the change in enclosure use over time, the lions used more of the enclosure as the study progressed. During Week 1, the lions were not observed in the new part of the enclosure, but were observed in that area with increasing frequency over time. This could partially be due to changed training and enrichment practices used in order to influence the lions' use of that area more often. Nonetheless, these husbandry practices may increase the lions' comfortability with that area and may lead to them using the new part of the enclosure more often on their own accord.

Similar to the time budget comparison, there was little difference between the lions' enclosure use during control nights and Sunset Safaris. The greater variety of zone use seen during Sunset Safaris may be related to multiple scatter feeds and addition of new enrichment during separate evenings, which caused increased movement through the enclosure. Zone 25, an area containing heated platforms for the lions and offering great viewing experience for visitors, was used more during control nights than during Sunset Safaris. Visitor sound levels and behavior (e.g., banging on the windows next to the heated rocks) may have influenced the lions to not spend much time there during Sunset Safaris.

SPI values demonstrate that the lions used the enclosure unevenly, which is supported by the charts separating enclosure use into original and new parts of the enclosure. However, the change in enclosure use over time suggests the lions may continue to spend more time in the new part of the enclosure. Overall SPI values were the same for Whipsnade Zoo and London Zoo. This uneven enclosure use reinforces the previously described idea of felids having core areas of their territory and has been similarly described in prides of wild lions [28].

### 4.3. Sociality

Both sociograms indicate that Heidi and Indi have a slightly stronger bond with Rubi than with each other, but differences in AI values are minimal. Schaller found that there was no consistent lioness leadership of an African lion pride [27]. However, because Asiatic lion prides typically are smaller than those of African lions [26], Indi and Heidi may look to Rubi for leadership as the eldest female of the pride, especially at Whipsnade Zoo when they may have been under increased stress after move. This may explain why Indi and Heidi both tended to associate more with Rubi than with each other. Nevertheless, a longer study would provide a more complete image of the sociality between the females, including more robust AI values to indicate their social preferences.

### 4.4. Personality

The profiles created from keeper questionnaires do not differ much between the lions, which was not the expected result. However, the interrater reliability results show that the method is reliable, as has been found in other studies [7, 16]. These ratings can be dependent on keeper experience with the animals and existing knowledge of animal personality. Interestingly, the range between trait ratings showing the most distinction between females did not correspond to level of keeper experience. For instance, the three keepers with the largest ranges between ratings for females had anywhere from the least to most experience with the lions. Additionally, it can be difficult to distinguish between the lionesses, which may have impacted the quality of the profiles. These questionnaires were completed in 2015 while the lions were at Whipsnade Zoo. Since then, the questionnaires have been expanded to 31 traits. Ideally, the questionnaires would be repeated again for more complete personality assessments.

The personality profiles created from behavioral observations were affected by having three subjects and a small data set, which makes the difference in trait ratings appear as large deviations in personality between the three females. Rather, these profiles may best be viewed as the lion that exhibited the most, least, or mid-amount of a trait. However, personality profiles from observations are a reliable and objective method that would be even more useful with a larger, long-term data set [11].

Previous events in the lions' lives may have greatly influenced the results of the personality questionnaires. In 2014, the females lost both of their parents within a few months. Shortly after this, they were transferred to Whipsnade Zoo. These experiences may have been traumatic for the lions and possibly have affected their behavior for an extended period of time. For example, the large amount of pacing behavior exhibited by the lions at Whipsnade may have been a response to these stressful events. As previously discussed, this pacing behavior may have carried over to the current study as a “scar” from these traumatic experiences [36].

Rubi had the highest average rating on keeper questionnaires for “Solitary,” but comparatively Heidi was the most solitary according to observation data. This may be connected to Heidi's high ratings in “Curious” and “Playful” in that she often investigated or played with objects. For instance, after the addition of new enrichment to the enclosure, Heidi spent more time interacting with the items compared to her siblings. Considering Heidi's time budget, she also exhibited more “Exploratory” behavior than her sisters. This increased time exploring and interacting with objects may indicate that she spent less time near her sisters, therefore increasing her rating for “Solitary.”

As expected considering the lions' time budgets, Indi had the highest rating for “Eccentric” on her profile created from observation data, which is due to her exhibiting the most stereotypic behavior. Before moving to Whipsnade Zoo, Rubi was the first lionesses to begin pacing after the loss of their parents. Heidi and Indi soon joined Rubi in this behavior, which then continued at Whipsnade Zoo. The fact that they followed Rubi in her display of stereotypic behavior may be an example of social facilitation and would support the aforementioned idea of Rubi as the leading female of the pride. Conversely, during observations at London Zoo, Indi often initiated the pacing behavior, and sometimes Rubi and/or Heidi would join her. Evidently, pride sociality plays a role in their behavioral patterns and preferences, but that role may be dynamic depending on their circumstances.

The personality profiles create opportunities for more individualized management of the lions, as demonstrated by Marieke Cassia and David's study on snow leopard personality [17]. They suggest that shier animals may need more places to hide, while bolder animals may benefit from increased enrichment opportunities. Heidi, as the most playful and curious of the three, may benefit from increased enrichment opportunities. Indi, as the most prone to eccentric behavior, may benefit from the same management strategies, but in order to decrease stereotypic behavior. Furthermore, with a longer study period and repeated questionnaires, it may be possible to determine which of the females would be the best option to breed, as previously demonstrated in cheetahs [2, 7].

These conclusions stem from observations and from the valuable perspectives of the keepers. In the past year, some of the keepers have spent more time with the lions and may be better able to distinguish personality differences between the females. Although personality is consistent overtime, the questionnaires may have been influenced by previous traumatic events and by keeper knowledge of the animals. Now that the lions have settled into Land of the Lions and the questionnaires have been expanded, it would be ideal to repeat the questionnaires.

## 5. Conclusions

1. This research provided valuable behavioral and personality profiles for the lionesses at London Zoo.
2. There was little difference in behavioral data between Sunset Safaris and control nights, which may be an indicator of little negative impact on the lions because of increased human social interaction.
3. The personality questionnaires were found to be a reliable method of assessing personality. The personality profiles created by keeper questionnaires showed little difference between the females, therefore making individual comparisons difficult. However, the profiles created from behavioral observations showed more of a distinction between the lions. Combined together, these profiles offer some opportunities for individualized management of the lions, including varied enrichment methods.
4. This research provides useful information for these specific lions to support current and future management decisions, and an interesting case study on individual animal adjustment to new environments.
5. A personality study of all captive Asiatic lions would enable a comparison of lion personality across a variety of captive management systems and further development of methodologies for felid personality research.

## Figures and Tables

**Table 1:** Members of the Asiatic lion pride at London Zoo.

Name	Age	Sex	Relationship
Rubi	7	F	Full siblings
Heidi	5	F	Full siblings
Indi	5	F	Full siblings
Bhanu	6	M	Unrelated

**Table 2:** Behavioral classes used to create time budgets. Individual behaviors come from the full ethogram, included in Table 10.

Class	Behaviors included
Inactive	Lie, sit, stand, stretch, stare

Locomotion	Walk, run, stalk, chase, climb, crouch

Stereotypic	Pace

Reproductive	Mount, sniff anogenital region, lordosis

Maintenance	Defecate, urinate, self-groom, scratch

Marking	Spray, scratch object

Vocalizations	Growl, grunt, roar, cough

Feeding	Eat, drink

Exploratory	Any interaction with objects, sniff, flehmen, dig

Interactions	Allogroom conspecific, bite conspecific, play with conspecific, chase conspecific, stalk conspecific, swat conspecific, head/body rub conspecific, tail up, band on glass

**Table 3:** Personality trait classes consisting of behaviors from full ethogram, included in Table 10.

Class	Behaviors included
Active	When an animal is exhibiting any observable behavior other than staring

Aggressive to conspecific	Bite conspecific, swat conspecific

Curious	Play with object, pounce on object, stalk object, swat object, bite object, dig, sniff, flehmen

Eccentric	Pacing

Friendly to conspecific	Allogroom conspecific, head/body rub conspecific, play with conspecific, tail up

Playful	Chase conspecific, play with conspecific/object, stalk conspecific/object, pounce on conspecific/object

Solitary	Time spent alone (i.e., greater than one body length away from conspecific)

Vocal	Growl, grunt, roar, cough

**Table 4:** Enclosure use values at London Zoo for each female and overall for each observation period, and in total for all observations.

	Daytime				Control nights				Sunset Safari				*All observation*
	Rubi	Heidi	Indi	*Overall*	Rubi	Heidi	Indi	*Overall*	Rubi	Heidi	Indi	*Overall*	
Zone 1	50.5%	45.5%	57.5%	*51.5%*	0.0%	1.3%	2.6%	*1.3%*	6.3%	1.4%	0.8%	*2.9%*	*27.8%*
Zone 2	9.2%	9.8%	23.4%	*14.2%*	6.7%	0.0%	0.0%	*2.2%*	6.6%	0.0%	0.0%	*2.3%*	*8.4%*
Zone 3	0.6%	1.3%	0.7%	*0.9%*	0.0%	0.4%	0.0%	*0.1%*	0.0%	0.0%	0.0%	*0.0%*	*0.5%*
Zone 4	0.2%	6.7%	0.4%	*2.4%*	0.9%	0.4%	1.3%	*0.9%*	1%	0.0%	0.0%	*0.4%*	*1.5%*
Zone 5	0.6%	0.4%	0.9%	*0.6%*	0.0%	0.0%	0.0%	*0.0%*	5.2%	0.3%	0.0%	*1.9%*	*0.8%*
Zone 6	1.7%	1.6%	0.0%	*1.1%*	0.0%	2.1%	0.0%	*0.7%*	1.7%	0.7%	0.0%	*0.8%*	*0.9%*
Zone 11	0.0%	0.0%	0.0%	*0.0%*	0.0%	0.0%	0.0%	*0.0%*	0.7%	0.0%	0.0%	*0.2%*	*0.1%*
Zone 12	4.1%	3.8%	2.9%	*3.6%*	13.4%	8.1%	0.4%	*7.2%*	27.3%	0.0%	0.0%	*9.4%*	*5.9%*
Zone 13	14.4%	14.4%	5.6%	*11.4%*	57.6%	51.3%	66.8%	*58.6%*	31.8%	61.6%	72%	*56.4%*	*33.0%*
Zone 14	2.2%	0.2%	0.0%	*0.8%*	0.0%	0.0%	0.0%	*0.0%*	0.3%	0.0%	0.0%	*1.9%*	*0.9%*
Zone 15	11.6%	4.4%	7.6%	*7.8%*	0.0%	0.0%	0.0%	*0.0%*	9.1%	5.1%	0.0%	*4.9%*	*5.3%*
Zone 16	0.0%	1.8%	0.0%	*0.6%*	0.0%	0.9%	0.0%	*0.3%*	0.3%	5.1%	0.0%	*1.9%*	*0.9%*
Zone 17	0.0%	0.0%	0.0%	*0.0%*	0.0%	0.0%	0.0%	*0.0%*	0.0%	0.0%	0.0%	*0.0%*	*0.0%*
Zone 18	0.7%	2.2%	0.2%	*1.0%*	0.0%	3.4%	0.4%	*1.3%*	0.3%	3.4%	0.8%	*1.6%*	*1.2%*
Zone 19	1.1%	2.5%	0.0%	*1.2%*	0.0%	1.7%	2.6%	*1.4%*	5.9%	2.1%	2.4%	*3.5%*	*1.9%*
Zone 20	0.4%	0.4%	0.0%	*0.2%*	0.0%	1.3%	0.9%	*0.7%*	0.3%	2.7%	3.1%	*2.0%*	*0.8%*
Zone 21	1.1%	1.8%	0.2%	*1.0%*	0.0%	3.8%	2.2%	*2.0%*	2.4%	5.1%	6.3%	*4.6%*	*2.2%*
Zone 22	0.2%	0.7%	0.0%	*0.3%*	0.0%	0.4%	2.2%	*0.9%*	0.3%	5.1%	0.8%	*2.2%*	*0.9%*
Zone 23	0.0%	0.2%	0.0%	*0.1%*	0.0%	0.0%	0.4%	*0.1%*	0.0%	0.7%	0.4%	*0.4%*	*0.2%*
Zone 24	1.1%	0.2%	0.0%	*0.4%*	0.4%	0.4%	0.4%	*0.4%*	0.0%	1.4%	0.0%	*0.5%*	*0.4%*
Zone 25	0.4%	1.1%	0.7%	*0.7%*	21.0%	24.4%	19.8	*21.7%*	0.0%	5.1%	7.5%	*4.1%*	*6.2%*

**Table 5:** Weekly enclosure use values for London Zoo daytime observations to demonstrate how the females' enclosure use changed throughout the study. Weeks 4 and 5 are combined as there were fewer observation sessions in Week 5.

	Week 1	Week 2	Week 3	Weeks 4-5
Zone 1	82.2%	52.5%	30.2%	54.1%
Zone 2	13.5%	19.2%	15.9%	7.9%
Zone 3	1.2%	0.9%	1.1%	0.4%
Zone 4	0.4%	1.1%	2.2%	5.3%
Zone 5	0.0%	0.4%	0.2%	1.4%
Zone 6	0.0%	0.2%	3.4%	0.4%
Zone 11	0.0%	0.0%	0.0%	0.0%
Zone 12	0.0%	0.0%	0.2%	12.1%
Zone 13	1.5%	21.6%	10.5%	7.7%
Zone 14	0.0%	0.0%	0.4%	2.2%
Zone 15	1.2%	0.2%	27.7%	0.0%
Zone 16	0.0%	0.0%	0.0%	2.0%
Zone 17	0.0%	0.0%	0.0%	0.0%
Zone 18	0.0%	1.5%	1.6%	0.6%
Zone 19	0.0%	0.7%	2.7%	1.2%
Zone 20	0.0%	0.0%	0.7%	0.2%
Zone 21	0.0%	0.9%	0.9%	1.8%
Zone 22	0.0%	0.0%	0.9%	0.2%
Zone 23	0.0%	0.0%	0.0%	0.6%
Zone 24	0.0%	0.2%	0.7%	0.6%
Zone 25	0.0%	0.7%	0.7%	1.2%

**Table 6:** Enclosure use values for each female and overall at Whipsnade Zoo in 2015.

	Rubi	Heidi	Indi	Overall
Zone 1	52.7%	76.7%	54.5%	61.5%
Zone 2	2.1%	0.9%	4.2%	2.5%
Zone 3	2.5%	1.9%	1.5%	1.9%
Zone 4	0.0%	0.9%	0.3%	0.4%
Zone 5	38.9%	14.5%	39.0%	30.6%
Zone 6	0.4%	0.0%	0.0%	0.1%
Zone 7	3.5%	5.0%	0.6%	3.0%
Zone 8	0.0%	0.0%	0.0%	0.0%

**Table 7:** SPI values for each female for Whipsnade Zoo and London Zoo observations.

	Whipsnade Zoo	Daytime	Control nights	Sunset Safari	*Overall 2016*
Rubi	0.69	0.78	0.87	0.70	*0.72*
Heidi	0.70	0.69	0.77	0.69	*0.63*
Indi	0.69	0.84	0.81	0.78	*0.74*

*Overall*	*0.69*	*0.76*	*0.81*	*0.67*	*0.69*

**Table 8:** Maximum, minimum, and average decibel levels for each observation period and overall.

	Max	Min	Average level
Daytime	85.9	37.7	63.2
Control nights	78.2	32.9	56.4
Sunset Safari	86.2	48.2	62.9

*Overall*	*86.2*	*32.9*	*60.8*

**Table 9:** Summary of keepers who completed lion personality questionnaires in 2015.

Keeper	Sex	Experience with these lions	Hours/week with the lions	Average range between ratings
1	M	6 months	25+	2.5
2	M	6 years	8	2.0
3	F	3.5 years	3	1.8
4	M	5 years	2	1.1
5	M	3 years	7	2.2
6	M	6 years	10	1.5
7	M	6 years	8	1.9

**(a):** 

State behaviour	Description
Out of sight (OOS)	Beyond one's range of vision
Decubitus–dorsal (DD)	Lays down on the dorsum
Decubitus–lateral (LD)	Lays down laterally
Decubitus–lateral–legs raised (DLLR)	Lays down laterally, one back leg raised
Decubitus–sternal (SD)	Lays down on the sternum
Sternal–sphynx (SPH)	Lays down on the sternum, back legs parallel and orientated forward
Sternal–lunula (LUN)	Lays down on the sternum, legs put to one side
Ears forward (EF)	Ears oriented forward
Ears backwards (EB)	Ears oriented backward
Facing conspecific (FC)	Stares at another animal of the same species
Facing observer (FO)	Stares at the observer
Facing public (FP)	Stares at the public
Proximity to conspecific–body length (BL)	Within one body length of other animal
Proximity to conspecific–far (F)	More than one body length away from the other animal
Proximity to conspecific–contact (C)	In body contact with conspecific
Sitting (SIT)	Upright position, all four feet on ground, front legs straight, back legs folded
Standing (STA)	Stands with all four legs extended, paws on the ground, immobile

**(b):** 

Event behaviour	Description
Allogroom (AG X) x is the animal	Licks the fur of a conspecific
Allogroomed (AGD b X)	Has the fur licked by a conspecific
Bare teeth (BAT X a) a for active	Animal opens its mouth and pulls the lips back, exposing its teeth
Receiving bare teeth (BAT X p) p for passive	Is on the receiving end of bared teeth
Bite (BT X)	Mouth closes on object or conspecific
Bitten (BT b X)	Is bitten by conspecific
Belly up (B UP)	Animal lies on its back with throat and belly exposed to the opponent
Belly up defensive posture (B UP DP)	Animal lies on its back with bared teeth, all four paws up with claws unsheathed
Chase (CH X)	Runs after conspecific or other being/object
Chased (CHD b X)	Pursued by conspecific
Climb up (CU)	Ascends an object or structure
Climb down (CD)	Descends an object or structure
Defensive open mouth (DOM X)	Mouth wide open in defensive stance
Drink (DR)	Lapps up water and swallows
Defecate (DF)	Relieves colon, releases faeces
Eat (EAT)	Ingests food by chewing and swallowing
Eat grass (EAG)	Ingest grass by chewing
Stretching (STR)	Extend all body and forelegs forward and put the back and tail up
Fight (F X)	Assaults conspecific
Assaulted (ASS b X)	Is assaulted by conspecific
Jump on (JM)	Attack suddenly and forcefully jump on the back of conspecific
Paw (PW)	Strike with the paw someone else
Flehmen (FH)	Sniffs, then lift head with open mouth, breath in, eyes almost closed and upper lip curled
Head butt (HB X)	Briefly pushes/bumps its head against a conspecific's head
Head butted (HB b X)	Has is head briefly bumped by a conspecific's head
Scratch (SRT)	Damage and mark the surface of by scraping with nails
Lick object (LO)	Protrudes tongue from the mouth and strokes object with it
Lick lips (LL)	Protrudes tongue from the mount and lick lips
Pace (PC)	Repetitive locomotion in a fixed pattern
Head shake (HSH)	Repetitive move of the head with short and quick movements
Circling (CIR)	Repetitive locomotion in a circle around
Twitch (TW)	Moving with a sudden, quick and short movements as reaction to something/someone
Move backwards (MB b X)	Moving backwards with ears backwards and head down as reaction to someone
Play object (PLO)	Interacts with objects
Play with conspecific (PL X a)	Initiates interaction with conspecific in a nonharmful manner (chasing, jumping, wrestling, etc.) and gets no response
Play with conspecific and is reciprocated (PL X)	Initiates interaction with conspecific in a nonharmful manner (chasing, jumping, wrestling, etc.) and gets some response
Played by conspecific (PL X p)	Passive receiver of conspecific play
Roll (RO)	Lying on the ground, the animal rotates its body from side to side; during the roll, the back is rubbed against ground, the belly is exposed and all paws are in the air
Rub–Body (RB)	Rubs body on conspecific or object
Rub–Head (RH)	Rubs head on conspecific or object
Rubbed (RBD)	Rubbed by a conspecific
Self-groom (SG)	Licks own fur
Sniff (SNF)	Smells by inhaling air through the nose
Spray (SP)	Stands with tail raised vertically and releases a jet of urine backwards against a vertical surface or object.
Stalk (STL)	Usually slow, forward locomotion with back and head slightly lowered and eyes focused on the stalked individual/object
Stare (STR)	Looks fixedly to something/someone
Tail up (TU)	Tail is held vertically, in a upright position
Tail slash (TS)	Standing or moving with tail bent over body, slashing
Tail tip (TT)	Prolonged, repeated movement of tip of the tail
Tail twitch (T TW)	A rapid flick of the tail in either a side to side or up to down motion
Urinate (U)	Releases urine, standing or squatting
Vocalization	Produces sounds or calls with is mouth/throat
Vocalization–chuff (CHF)	Cat expels jets of air through the nose creating a low-intensity, soft, pulsed sound, described as being similar to the snorting of a horse
Vocalization–grunt/cough (GRT)	Short, throaty call, characterized by the deep contraction and expansion of the diaphragm
Vocalization–growl (GRL)	A low-pitched, throaty, rumbling noise produced while the mouth is closed
Vocalization–hiss (HS)	A drawn-out, low-intensity hissing sound produced by rapid expulsion of air from the cat's mouth, usually during exhalation
Vocalization–roar (RO)	Long, throaty, high intensity call
Vocalization–syndetic call (SC)	Amiable call with the purpose of gather or appease conspecifics
Walk (WK)	Forward locomotion at a slow gait
Run (RU)	Forward locomotion at a quick gait
Warning bite (W BT X)	Snap teeth in response to an unwelcomed closing individual
Yawn (YN)	The mouth is opened widely, the head tips back, lips are pulled back so that the teeth are exposed
Look around (LOA)	Turn one's eyes toward something or in some direction in order to see
Crouch (CR)	Bend close to the ground or stoop low for lay down
Crouch for other lion (CR X)	Stoop low and lays down on the sternum with ears backwards, head down or open mouth for submit to someone
Dive in (DIN)	Plunge into water and stay in the water
*Breeding behaviours*	
Mount (MT)	Moves on top of conspecific in the attempt to copulate
Nape bite (N BT)	The male performs an inhibited nape bite, where he will place his mouth on or around the back of the female's neck at the moment of, or just after, ejaculation, but is unlikely to actually bite down
Being mounted (BM)	Is mounted by other lion
Sniff anogenital (SNA)	Smells the anogenital region of conspecific

**Table 11:** Zone descriptions of London zoo.

Zone	Features	Approx. % of total section area
	*Females' section: area = 1395 m* ^*2*^	
1	Back right corner of enclosure; next to entrance to indoor area; borders raised walkway along back wall	4.85
2	Front right corner; contains chain-link fence next to public walkway	4.85
3	Borders raised walkway along back wall; next to entrance to indoor area	3.88
4	Surrounds wooden platform	4.85
5	Borders raised walkway along back wall; includes metal gate to male's section of the enclosure	3.88
6	Front left corner of original area of enclosure; contains small covered area under rock wall	3.88
7–10	Located indoors	N/A
11	Lower level of wooden platform	1.46
12	Mid-level of wooden platform; often used to climb up to Zone 13	0.97
13	Top level of the wooden platform; offers high viewpoint	1.46
14	Top of a concrete slab in front of the entrance to indoor area	1.46
15	Area underneath Zone 14	1.46
16	Grass-covered platform in front of Zone 6; overlooks moat	0.97
17	Located under Zone 16	0.97
18	Thin zone bordering edge of moat	8.74
19	Start of new area of enclosure; contains rocky ledge along back wall	6.80
20	Covers right side of the 360 area; right side looks over the moat	4.85
21	Contains section of trees and bushes	17.48
22	Covers left side of 360 area	8.74
23	Back left corner of new area of enclosure	4.85
24	Allows access to Zone 25	8.74
25	Covered area containing heated platforms (“Hot rocks”); where training occurs	4.85

	*Male's section: area = 800 m* ^*2*^	
26	Faces access area where staff often walk; where outdoor training occurs	15.09
27	Contains access door for indoor area	9.43
28	Also faces access area where staff walk; contains part of small hill in middle of enclosure	20.75
29	Contains old train car/boxes; borders raised walkway	26.42
30	Borders mongoose enclosure	13.21
31	Contains train car where feeding sometimes occurs; allows access to train station platform with large public viewing windows	15.09

**Table 12:** Zone descriptions of Whipsnade zoo.

Zone	Features	% of Total area
	*Area = 230 m* ^*2*^	
1	Back right corner; away from walkways	24.24
2	Front right corner; contains sleeping platform; walkway along front edge	22.73
3	Back left corner; walkway bordering side edge	24.24
4	Front left corner; walkway along front and side edges; contains training platform	22.73
5	Sleeping platform	1.52
6	Training platform	1.52
7	Area underneath sleeping platform	1.52
8	Area underneath training platform	1.52
